# Photosynthetic Characteristics and Chloroplast Ultrastructure of Summer Maize Response to Different Nitrogen Supplies

**DOI:** 10.3389/fpls.2018.00576

**Published:** 2018-05-01

**Authors:** Zheng Liu, Jia Gao, Fei Gao, Peng Liu, Bin Zhao, Jiwang Zhang

**Affiliations:** State Key Laboratory of Crop Biology, College of Agronomy, Shandong Agricultural University, Tai’an, China

**Keywords:** summer maize, nitrogen rate, grain yield, photosynthetic characteristics, chloroplast ultrastructure

## Abstract

Maize (*Zea mays* L.) is the important crop over the world. Nitrogen (N) as necessary element affects photosynthetic characteristics and grain yield of summer maize. In this study, N0 (0 kg N ha^-1^), N1 (129 kg N ha^-1^), N2 (185 kg N ha^-1^), and N3 (300 kg N ha^-1^) was conducted using hybrid ‘ZhengDan958’ at Dawenkou research field (36°11′N, 117°06′E, 178 m altitude) in the North China Plain to explore the effects of N rate on photosynthetic characteristics and chloroplast ultrastructure. Gas exchange parameters, chlorophyll fluorescence parameters, leaf area index (LAI), chlorophyll SPAD value, chloroplast ultrastructure, dry matter weight and grain yield were measured. At physiological maturity stage, dry matter weight and grain yield of N2 increased by 33–52% (*P* ≤ 0.05) and 6–32% (*P* ≤ 0.05), respectively, compared with other treatments. During the growing from silking (R1) to milk (R3) stage, LAI of N0 and N1 were 35–38% (*P* ≤ 0.05) and 9–23% (*P* ≤ 0.05) less than that of N2, respectively. Chlorophyll SPAD value of N0 and N1 were 13–22% (*P* ≤ 0.05) and 5–11% (*P* ≤ 0.05) lower than that of N2. There was no significant difference in LAI and chlorophyll SPAD value between N2 and N3 during the period from R1 to R3 (*P* > 0.05). The net photosynthetic rate (*P*_n_), maximal quantum efficiency of PSII (*F*_v_/*F*_m_) and quantum efficiency of PSII (Φ_PSII_) were higher with the increase of N rate up to N2 (*P* ≤ 0.05), and those of N3 were significantly less than N2 (*P* ≤ 0.05). In compared with N2, the chloroplast configuration of N0 and N1 became elliptical, almost circular or irregular. The membrane of chloroplast and thylakoid resolved with growing stage, and the number of chloroplast per cell and lamellae per grana decreased under N0 and N1 treatment (*P* ≤ 0.05). Under N0 and N1 treatments, summer maize had more negative photosynthetic characteristics. The more number of osmium granule and vesicle and the larger gap between lamellae were shown in N3. Therefore, N2 treatment, 185 kg N ha^-1^, is the appropriate application rate for grain yield, photosynthesis and chloroplast ultrastructure.

## Introduction

Nitrogen (N) is not only necessary element but also the limiting factor for grain yield of summer maize (*Zea mays* L.). However, the irrational N rate hinder production increase and result in environmental pollution (Zhang et al., 2016; Kong et al., 2017). In the North China Plain (NCP), local farmers’ traditional N rate is 300 kg N ha^-1^ to obtain higher grain yield, which is 62% more that the recommended N rate (185 kg N ha^-1^), and the continued increases in N rate have not resulted in the commensurate increases in summer maize yield (Jin et al., 2012; Zhu et al., 2014; Liu et al., 2017).

Previous studies indicate that fertilizers, especially N fertilizer, make over 50% contribution in grain yield increase (Tilman et al., 2011; Fan et al., 2012). N managements promote maize growth and achieve high yield. The excessive N fertilizer may cause the reduction yield of summer maize whilst harming environment (Cui et al., 2010). The dry matter weight and grain yield are closely related. The basis of grain yield is high dry matter weight, especially accumulation of dry matter after anthesis (Plénet and Lemaire, 1999; Rajcan and Tollenaar, 1999). N application significantly increase dry matter weight, but excessive N rate would delay crops maturity and inhibit N translocation from vegetative organs to grain which is not conductive to crop production (Fang et al., 2006; Zhao et al., 2006). Leaf area index (LAI) is used to evaluate the development and structure of canopy. N deficiency reduce LAI since the leaves become slender, which result in light-leaking loss (Wilhelm et al., 2000; Bavec and Bavec, 2002). Excessive N rate make vegetative organs vigorously grow resulting in self-shade within the population, which have a negative effect on crop production. The chlorophyll SPAD value is used to determine the effect of N rate on leaf chlorophyll concentration since there is a non-linear relationship between them (Richardson et al., 2002). The chlorophyll SPAD value determine the N status of summer maize easily and quickly. N is the constituent of chlorophyll and N deficiency reduce leaf chlorophyll concentration. The parameters of leaf gas exchange and chlorophyll fluorescence reflect the carbon assimilation performance of the photosynthetic mechanism (Scheuermann et al., 1991; Nissanka et al., 1997; Fromm and Fei, 1998). The leaves suffer stomatal closure and related enzyme degradation under N deficiency, which decrease photosynthetic rate and PSII photochemical efficiency. The above negative performance result in significant decreases in grain yield. The changes in photosynthetic characteristics and chloroplast ultrastructure of field-grown summer maize would be responsible for the decreased yield with inaptitude N supply.

The morphology and ultrastructure of chloroplast directly affect photosynthesis, and have significant effects on dry matter weight and grain yield of summer maize (Slack et al., 1969; Chotewutmontri and Barkan, 2016; Shao et al., 2016). The intact chloroplast membrane is essential for the physiological function of chloroplasts; thylakoid membranes as the attachment site of chlorophyll are the biochemical reaction sites (Hall et al., 1972; Majeran et al., 2008). Previous studies showed that the concentration of N significantly affects the morphology and function of chloroplast (Harel et al., 1977; Tóth et al., 2002; Salesse-Smith et al., 2017). Adverse circumstances lead to chloroplast membrane and thylakoid membrane folds, thereby undermining its physiological function (Ristic and Cass, 1992). Maize, a C4 plant, generate C4 compounds catalyzed by phosphoenolpyruvate carboxylase (PEPC) in chloroplasts in mesophyll cells; these C4 compounds produce glucose through the Calvin cycle in the vascular sheath cells. Environmental stress impairs the morphology and function of chloroplasts, which weakens photosynthesis and decreases grain yields (Caers et al., 1985; Kratsch and Wise, 2000; Xu et al., 2006).

In comparison to the information on grain yield, dry matter weight, N use efficiency, and root growth and development responses (Jin et al., 2012; Zhu et al., 2014; Liu et al., 2017) only limited knowledge exists on the response of chloroplast ultrastructure to N rate under field conditions. Another interesting question is why does grain yield not increase continually with the increases in N rate. In order to answer above questions, this study was conducted in the field from 2015 to 2016 to evaluate the effects of N rate on photosynthetic characteristics and chloroplast ultrastructure of summer maize.

## Materials and Methods

### Experiment Site

**FIGURE 1:**
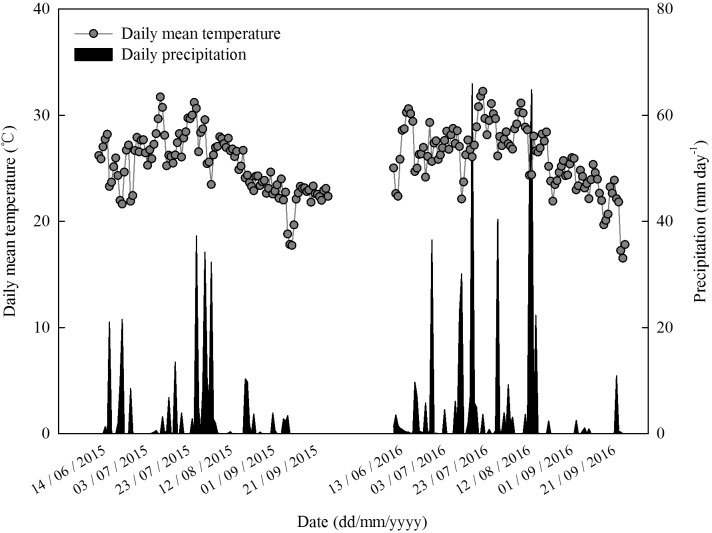
Daily mean air temperature and precipitation during the maize growing seasons in 2015 and 2016 at Dawenkou research field (36°11′N, 117°06′E, 178 m a.s.l.).

The experiment was conducted at Dawenkou research field (36°11′N, 117°06′E, 178 m a.s.l.) and State Key Laboratory of Crop Science in 2015 and 2016. This research field which is located in Shandong Province, China is characterized by brown loam soils and a temperate continental monsoon climate. Daily mean temperature and precipitation during the study period growing seasons are presented in **Figure 1**.

### Experiment Design

Summer maize (*Z. mays* L. ‘ZhengDan958’), the most popular hybrid in China, was planted at 7.5 × 10^4^ seeds ha^-1^ on June 14th, 2015 and June 13th, 2016, and harvested on 1st October. The growing period of 2 years was listed in **Table 1**. Each plot consisted of 10 rows of maize planted 0.6 m apart and 40 m long. Four N rate (0, 129, 185, and 300 kg ha^-1^) were designated as four treatment (N0, N1, N2, and N3). The local N application rate was 300 kg ha^-1^, whereas the recommended N rate based on previous research was 185 kg ha^-1^. A rate of 129 kg ha^-1^, 30% less than the recommendation, was chosen to provide N stress. N applications were split between pre-planting, six-leaf stage (V6), and silking stage (R1) (**Table 2**). Phosphorus (P) and potassium (K) applied to each treatment were 56 kg P ha^-1^ and 131 kg K ha^-1^ as calcium superphosphate and potassium chloride. P was applied 30 kg P ha^-1^ before seeding and 26 kg P ha^-1^ at V6. The K application was split-applied, 30, 71, and 30 kg K ha^-1^ before seeding, at V6, and at R1. All fertilizer was applied by furrow between rows. Irrigation (80 mm) was applied at seeding only. In all experimental fields, weeds were well controlled by intermixture of nicosulfuron and atrazine. The straw derived from previous crops, winter wheat, was returned by rotary tillage before planting summer maize. The 50% phoxim emulsion (3 kg ha^-1^) was applied by furrow between rows while seeding. No obvious water or pest stress occurred during the growing season.

**Table 1:** Growing period of summer maize in 2015 and 2016.

Year	Seeding	V6	V12	R1	R3	R6
2015	15th June	13th July	1st August	13th August	11th September	1st October
2016	13th June	11th July	31st July	10th August	10th September	1st October


*The criterion for growth stage is that more than 50% plants of population reached this stage. V6: six-leaf stage; V12: twelve-leaf stage; R1: silking stage; R3: milk stage; R6: physiological maturity stage.*

**Table 2:** Nitrogen timing (V6: six-leaf stage; R1: silking stage) and application rate (kg ha^-1^) for different N treatments applied to summer maize.

Treatment	Before seeding	V6	R1
N0	–	–	–
N1	21	63	45
N2	30	90	65
N3	50	145	105


*Nitrogen applied to maize are 0 kg N ha^-*1*^ (N0), 129 N ha^-*1*^ (N1), 185 kg N ha^-*1*^(N2), and 300 kg N ha^-*1*^ (N3).*

### Grain Yield and Its Components

At R6, 30 ears were obtained from the center three rows of each plot to determine kernel number per ear and thousand-kernel weight. Ears ha^-1^ was determined by counting plants per plot. All the kernels were air-dried to investigate yield, and grain yield was expressed at 14% moisture (Gb/T 29890-2013, 2013). Grain yield was calculated as: Grain yield (Mg ha^-1^, at about 140 g kg^-1^ moisture) = ear number per hectare × kernel number per ear × thousand kernel weight/10^9^.

### Leaf Area Index (LAI)

Fifteen representative plants were marked in each plot at V6, 12-leaf stage (V12), R1, R3 and physiological maturity stage (R6) to measure maximum width (λ_m_), total length (*L*_t_), width at ligule level (λ_0_) and distance from ligule to the point of maximum width (*L*_x_) of each leaf for the individual tagged plants. The leaf area was calculated according to the method of Combe (2005), and leaf area index (LAI) is the leaf area of the unit land area.

### Chlorophyll SPAD Value

Ten plants were randomly selected from each plot at V6, V12, R1, R3, and R6 from 10:00 AM to 1:00 PM. SPAD value was measured non-destructively with the portable SPAD-502 Chlorophyll Meter (Minolta Camera Co., Japan) from the functional leaf of each plant, and then averaged. During measurements with the SPAD-502, the sensor head was shaded with the operator’s own body as recommended by the manufacturer to avoid direct sunlight from reaching the instrument.

### Dry Matter Weight

Five plant samples were obtained from the center of each plot at V6, V12, R1, R3, and R6, and separated into grain and straw at R6. The samples were dried in an oven (DHG-9420A; Bilon Instruments Co. Ltd., Shanghai, China) at 85 ± 5°C, after heating at 105°C for 30 min, to a constant weight and the dry weight was then measured.

### Leaf Gas Exchange Parameters and Chlorophyll Fluorescence Parameters

At V6, R1 and milk stage (R3), the photosynthetic rate (*P*_n_), transpiration rate (*T*_r_), stomatal conductance (*G*_s_) and intercellular CO_2_ concentration (*C*_i_) of the functional leaf (last ligulated leaf at V6, and ear leaf at R1 and R3) (Escobar-Gutiérrez and Combe, 2012) were measured using a portable infrared gas analyzer (CIRAS II, PP System, Hansatech, United Kingdom) from 10:00 AM to 1:00 PM. Measurement conditions were kept consistent: LED light source and the PAR was 1600 μmol m^-2^. CO_2_ concentration was maintained at a constant level of 360 μmol mol^-1^ using a CO_2_ injector with a high-pressure liquid CO_2_ cartridge source (Wang et al., 2009; Qi et al., 2012).

Chlorophyll fluorescence was measured with a FMS-II plus modulated fluorometer (Hansatech, United Kingdom) on the same leaves as used for gas exchange measurements from 10:00 AM to 1:00 PM. Minimal fluorescence (*F*_0_) was measured under a weak pulse of modulating light over a 0.8 s period, and maximal fluorescence (*F*_m_) was induced by a saturating pulse of light (8000 μmol m^-2^ s^-1^) applied over 0.8 s. The maximal quantum efficiency of PSII was determined as *F*_v_/*F*_m_, where *F*_v_ is the difference between *F*_0_ and *F*_m_. An actinic light source (600 μmol m^-2^ s^-1^) was then applied to achieve steady-state photosynthesis and to obtain *F*_s_ (steady-state fluorescence yield), after which a second saturation pulse was applied for 0.7 s to obtain *F*_m_′ (light-adapted maximum fluorescence). Fluorescence parameters were calculated by FMS-II, based on the dark-adapted for 20 min and light-adapted fluorescence measurements (Wang and Jin, 2005; Wang et al., 2009). The quantum efficiency of PSII (Φ_PSII_) was calculated as (*F*_m_′ -*F*_s_)/*F*_m_′ (Genty et al., 1989).

### Transmission Electron Microscope (TEM) Sample Preparation and Observation

In 2016, the illuminated sides of five ear leaves were obtained from the center of each plot at V6, R1, and R3 stages. A square section of a leaf (0.5 cm × 0.5 cm) near the center vein of each leaf was removed with a blade. After fixation with 2.5% glutaraldehyde for 4 h, leaf cells were post-fixed with osmic acid at 4°C for 4 h and then dehydrated with ethanol. When embedded in spurr resin at 70°C for 8 h, thin sections were cut from leaf samples with an LKB-V ultra-microtome (Pharmacia LKB Co., Sweden) and placed upon 250 mesh grids. Samples were double stained using stem uranyl acetate and lead citrate and then observed and randomly photographed using a Hitachi-600 transmission electron microscope (Hitachi Ltd., Japan).

### Statistical Analysis

Data of chloroplast ultrastructure were tested by Shapiro–Wilk and Levene tests and then analyzed by one-way analysis of variance (one-way ANOVA) procedure using SPSS 17.0 software (SPSS Inc., United States) with *P* ≤ 0.05 considered significant. Two-way repeated measures analysis of variance (two-way repeated ANOVA) with Bonferroni was used compare other data after Shapiro–Wilk and Mauchly’s tests. Treatments were compared with Duncan’s multiple range test (*P* ≤ 0.05).

## Results

### Grain Yield and Its Components

As shown in **Table 3**, N rate had extremely significant effects on grain yield and its components of summer maize (*P* ≤ 0.01). Grain yield increased with the increase of N rate up to N2, and no greater yield was obtained in N3. Compared with N0, grain yield of N1 and N2 increased by 22% (*P* ≤ 0.05) and 32% (*P* ≤ 0.05) in 2015, by 16% (*P* ≤ 0.05) and 29% (*P* ≤ 0.05) in 2016, respectively. In 2015, N2 and N3 did not differ (*P* > 0.05). In 2016, N3 was 6% (*P* ≤ 0.05) less than N2. Ears per hectare, kernels per ear and thousand-kernel weight (TKW) of N0 were the lowest (*P* ≤ 0.05). There was no significant difference in ears per hectare between N2 and N3 (*P* > 0.05). The kernels per ear of N0 was significantly lower than others (*P* ≤ 0.05) and no significant difference was obtained among N1, N2, and N3 (*P* > 0.05). TKW of N2 increased by 7% (*P* ≤ 0.05) and 4% (*P* ≤ 0.05) in 2015, by 10% (*P* ≤ 0.05) and 9% (*P* ≤ 0.05) in 2016, respectively, compared with N0 and N1. In 2015, TKW between N2 and N3 did not differ (*P* > 0.05) but that of N3 decreased by 5% (*P* ≤ 0.05) compared with N2 in 2016.

**Table 3:** Grain yield and yield components of summer maize as affected by N rate.

Year	Treatment	Grain yield	Ears	Kernels	TKW
		(Mg ha^-1^)	(No. 10^4^ ha^-1^)	(No. ear^-1^)	(g)
2015	N0	10.1^c^	6.7^c^	490.1^b^	307.9^c^
	N1	12.3^b^	7.0^b^	553.2^a^	318.0^b^
	N2	13.3^a^	7.4^a^	544.9^a^	330.9^a^
	N3	13.4^a^	7.4^a^	552.1^a^	327.0^a^
2016	N0	11.2^d^	6.9^b^	519.9^b^	312.1^c^
	N1	13.0^c^	7.4^a^	554.8^a^	316.0^c^
	N2	14.4^a^	7.4^a^	567.1^a^	343.1^a^
	N3	13.5^b^	7.4^a^	559.9^a^	327.0^b^
Shapiro–Wilk test		NS	NS	NS	NS
Mauchly’s test		NS	NS	NS	NS
Two-way repeated ANOVA					
Treatment (T)		^∗∗^	^∗∗^	^∗∗^	^∗∗^
Year (Y)		^∗^	NS	^∗^	^∗^
T × Y		NS	NS	NS	NS


*TKW, thousand-kernel weight. Values followed by a different small letter in the same year are significantly different at P ≤ 0.05 with Duncan’s multiple range test. NS, not significant, P > 0.05. ^∗^, ^∗∗^ significant at the 0.05 and 0.01 probability level, respectively. Nitrogen applied to maize are 0 kg N ha^-*1*^ (N0), 129 N ha^-*1*^ (N1), 185 kg N ha^-*1*^ (N2), and 300 kg N ha^-*1*^ (N3).*

### Leaf Area Index (LAI)

**FIGURE 2:**
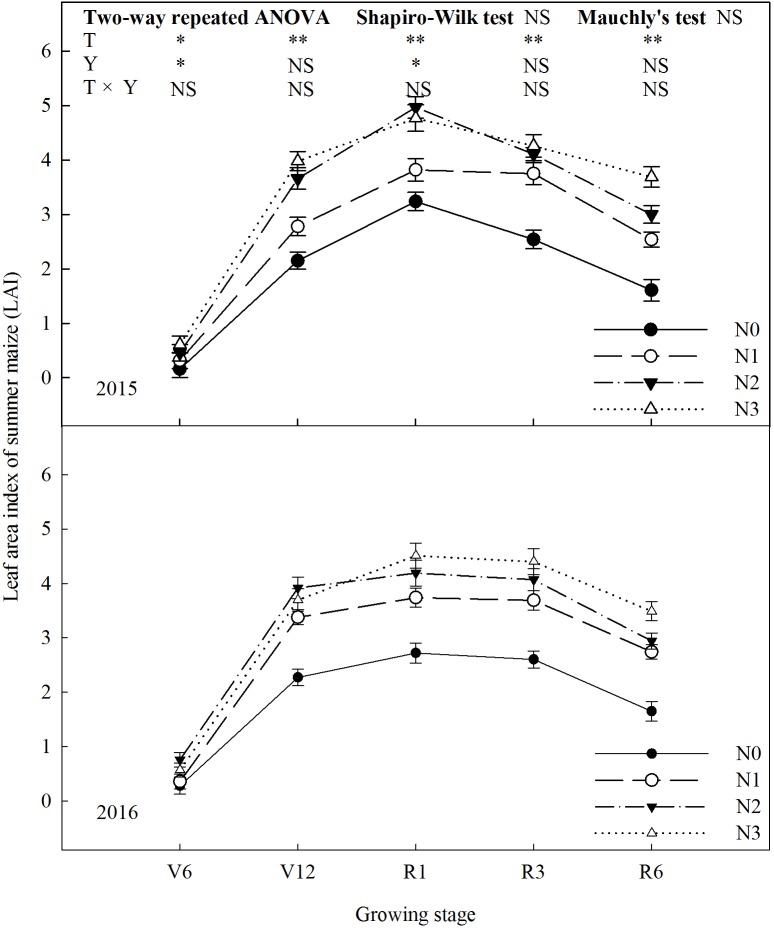
Effect of nitrogen rate on leaf area index (LAI) of summer maize during growth stages in 2015 and 2016. V6: six-leaf stage, V12: twelve-leaf stage, R1: silking stage, R3: milk stage, R6: physiological maturity stage. Nitrogen applied to maize are 0 kg N ha^-1^ (N0), 129 N ha^-1^ (N1), 185 kg N ha^-1^ (N2), and 300 kg N ha^-1^ (N3). Error bars are given as SD. NS, not significant, *P* > 0.05. ^∗^, ^∗∗^Significant at the 0.05 and 0.01 probability level, respectively.

N fertilization promoted LAI of summer maize (**Figure 2**). LAI of N2 was significantly higher than that of N0 and N1 during the growing season (*P* ≤ 0.05). Take 2015 as an example, LAI of N2 increased by 53% (*P* ≤ 0.05) and 30% (*P* ≤ 0.05) compared with N0 and N1 at R1. During the period from V6 to R3, N2 and N3 did not differ (*P* > 0.05). At R6, LAI of N3 increased by 23% (*P* ≤ 0.05) compared with N2. LAI decreased significantly by 37% for N0, 32% for N1, 27% for N2 and 13% for N3, respectively, from R3 to R6. The trend in 2016 was similar to 2015 (the effect of interaction of treatment and year was not significant, *P* > 0.05).

### Chlorophyll SPAD Value

**FIGURE 3:**
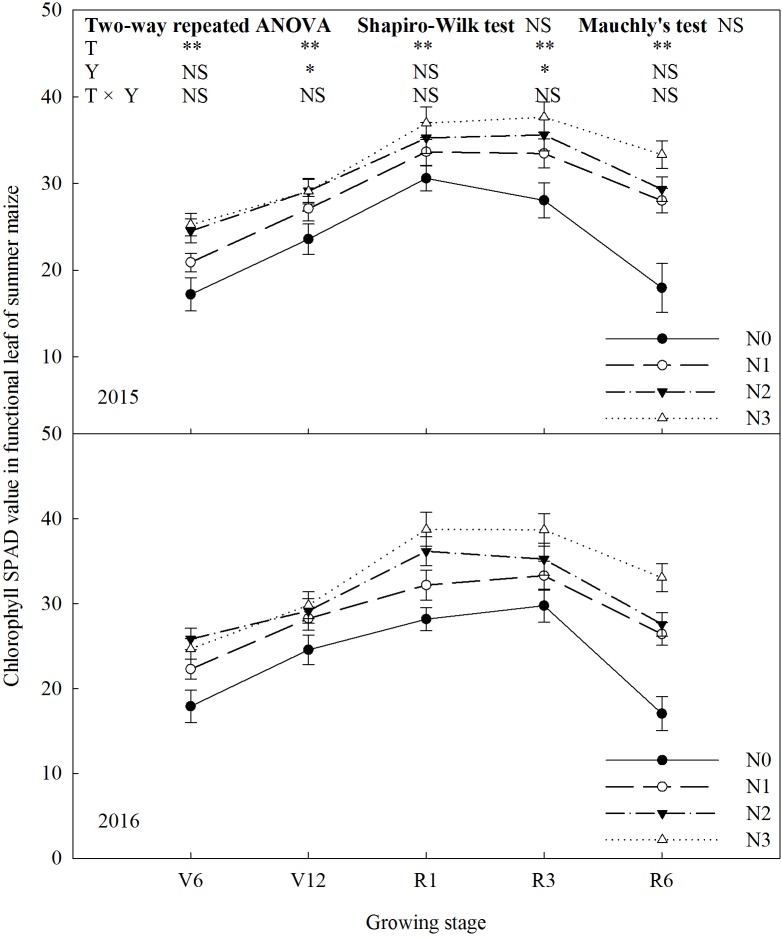
Effect of nitrogen rate on chlorophyll SPAD value in functional leaves of summer maize during growth stages in 2015 and 2016. V6: six-leaf stage, V12: twelve-leaf stage, R1: silking stage, R3: milk stage, R6: physiological maturity stage. Nitrogen applied to maize are 0 kg N ha^-1^ (N0), 129 N ha^-1^ (N1), 185 kg N ha^-1^ (N2), and 300 kg N ha^-1^ (N3). Functional leaf: last ligulated leaf at V6, and ear leaf at other stages. Error bars are given as SD. NS, not significant, *P* > 0.05. ^∗^, ^∗∗^Significant at the 0.05 and 0.01 probability level, respectively.

Chlorophyll SPAD value increased significantly (*P* ≤ 0.01) with N rate (**Figure 3**). In 2015, chlorophyll SPAD value of N2 was 43% (*P* ≤ 0.05) and 17% (*P* ≤ 0.05) higher than that of N0 and N1 at V6, and N2 and N3 did not differ (*P* > 0.05). At V12, there was no significant difference among treatments, except N0. From R1 to R3, chlorophyll SPAD value was basically stable. At R6, chlorophyll SPAD value of N3 increased by 86% (*P* ≤ 0.05) compared with N0, 14–19% (*P* ≤ 0.05) compared with N1 and N2, and there was no significant difference between N1 and N2 (*P* > 0.05). During the period from R3 to R6, chlorophyll SPAD value decreased significantly by 40% for N0, 16% for N1, 17% for N2 and 11% for N3. The trends for 2 years were similar (*P* > 0.05).

### Dry Matter Weight

**FIGURE 4:**
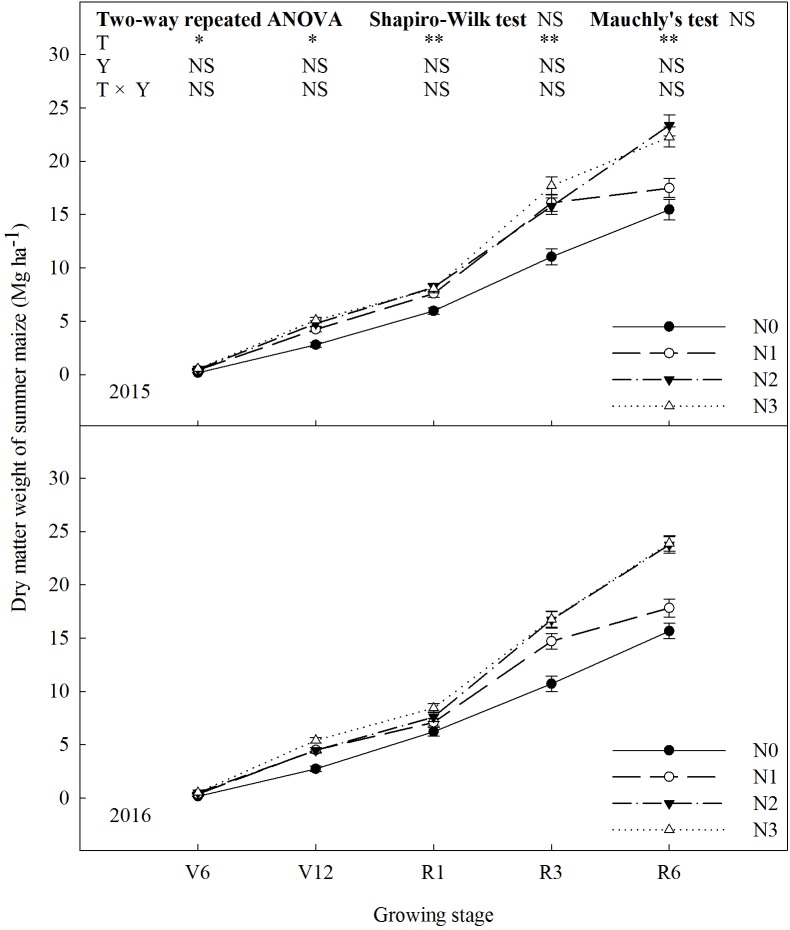
Effect of nitrogen rate on dry matter weight of summer maize during growth stages in 2015 and 2016. V6: six-leaf stage, V12: twelve-leaf stage, R1: silking stage, R3: milk stage, R6: physiological maturity stage. Nitrogen applied to maize are 0 kg N ha^-1^ (N0), 129 N ha^-1^ (N1), 185 kg N ha^-1^ (N2), and 300 kg N ha^-1^ (N3). Error bars are given as SD. NS, not significant, *P* > 0.05. ^∗^, ^∗∗^Significant at the 0.05 and 0.01 probability level, respectively.

N rate affected dry matter weight of summer maize significantly (**Figure 4**). The dry matter weight increased with increase in N rate up to N2 (*P* ≤ 0.05). N2 and N3 did not differ in dry matter weight (*P* > 0.05). At R1, dry matter weight of N2 increased by 37% (*P* ≤ 0.05) and 8% (*P* ≤ 0.05) compared with N0 and N1, and there was no significant difference between N2 and N3 (*P* > 0.05). At R6, dry matter weight of N2 was 51% (*P* ≤ 0.05) and 34% (*P* ≤ 0.05) higher than that of N0 and N1. The results of 2 years did not differ (*P* > 0.05).

### Leaf Gas Exchange Parameters

**FIGURE 5:**
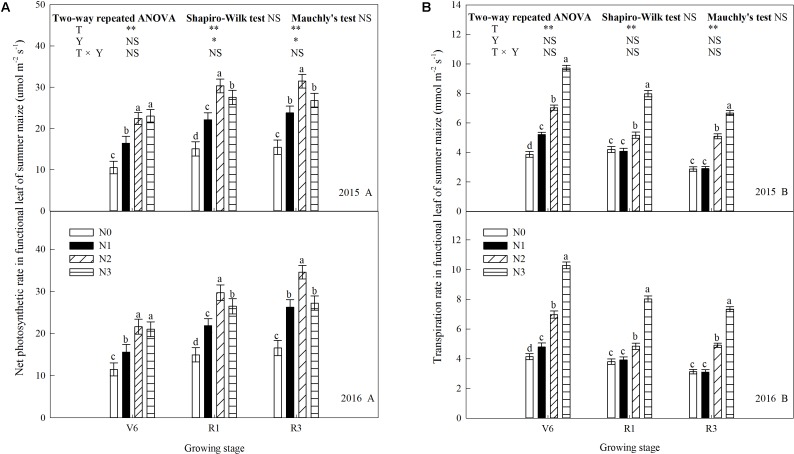
Effect of nitrogen rate on photosynthetic rate (*P*_n_) and transpiration rate (*T*_r_) in functional leaves of summer maize during growth stages in 2015 and 2016. **(A)** Photosynthetic rate, **(B)** transpiration rate. V6: six-leaf stage, R1: silking stage, R3: milk stage. Nitrogen applied to maize are 0 kg N ha^-1^ (N0), 129 N ha^-1^ (N1), 185 kg N ha^-1^ (N2), and 300 kg N ha^-1^ (N3). Functional leaf: last ligulated leaf at V6, and ear leaf at R1 and R3. Treatments in the same growing stage with different small letters are significantly different at 5% probability level. Error bars are given as SD. NS, not significant, *P* > 0.05. ^∗^, ^∗∗^Significant at the 0.05 and 0.01 probability level, respectively.

**FIGURE 6:**
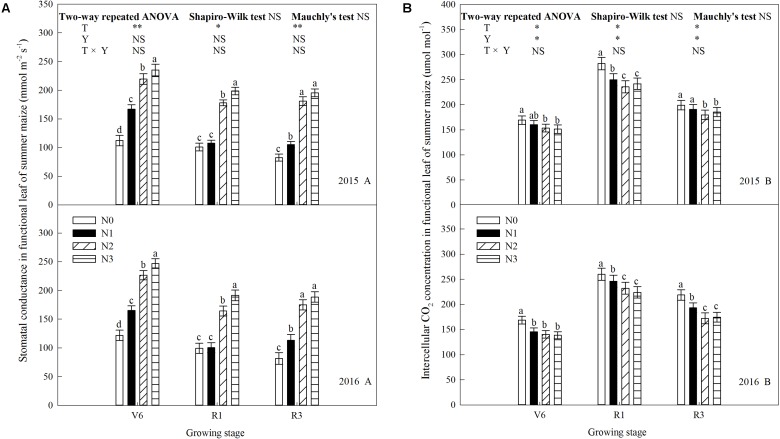
Effect of nitrogen rate on stomatal conductance (*G*_s_) and intercellular CO_2_ concentration (*C*_i_) in functional leaves of summer maize during growth stages in 2015 and 2016. **(A)** Stomatal conductance, **(B)** intercellular CO_2_ concentration. V6: six-leaf stage, R1: silking stage, R3: milk stage. Nitrogen applied to maize are 0 kg N ha^-1^ (N0), 129 N ha^-1^ (N1), 185 kg N ha^-1^ (N2), and 300 kg N ha^-1^ (N3). Functional leaf: last ligulated leaf at V6, and ear leaf at R1 and R3. Treatments in the same growing stage with different small letters are significantly different at 5% probability level. Error bars are given as SD. NS, not significant, *P* > 0.05. ^∗^, ^∗∗^Significant at the 0.05 and 0.01 probability level, respectively.

N rate affected leaf gas exchange parameters significantly (*P* ≤ 0.05) and the trends of 2 years’ results were similar (*P* > 0.05). As shown in **Figure 5A**, net photosynthetic rate (*P*_n_) increased (*P* ≤ 0.05) with increase N rate up to N2 and decreased (*P* ≤ 0.05) under N3 (except V6). During the period from V6 to R3, *P*_n_ gradually increased. At V6, *P*_n_ of N2 increased by 113% (*P* ≤ 0.05) and 36% (*P* ≤ 0.05) compared with N0 and N1, respectively. N2 and N3did not differ (*P* > 0.05). At R1, *P*_n_ of N2 increased by 101% (*P* ≤ 0.05), 37% (*P* ≤ 0.05), and 10% (*P* ≤ 0.05) compared with N0, N1, and N3. The trend at R3 was accord with that at R1. Transpiration rate (*T*_r_) increased with N rate and decreased with growing stage (**Figure 5B**). At V6, *T*_r_ of N3 was 152% (*P* ≤ 0.05), 86% (*P* ≤ 0.05), and 38% (*P* ≤ 0.05) higher than that of N0, N1, and N2, respectively. The similar trends occurred at R1 and R3, however, N0 and N1 did not differ (*P* > 0.05). Stomatal conductance (*G*_s_) increased with N rate, however, N0 and N1 did not differ at R1 and N2 and N3 did not differ at R3 (**Figure 6A**). As shown in **Figure 6B**, N fertilization reduced intercellular CO_2_ concentration (*C*_i_) significantly. *C*_i_ increased and then decreased during growth stages. *C*_i_ at R1was significantly higher than that at other times. *C*_i_ of N0 was the highest (*P* ≤ 0.05), and N2 and N3 did not differ (*P* > 0.05).

### Chlorophyll Fluorescence Parameters

**FIGURE 7:**
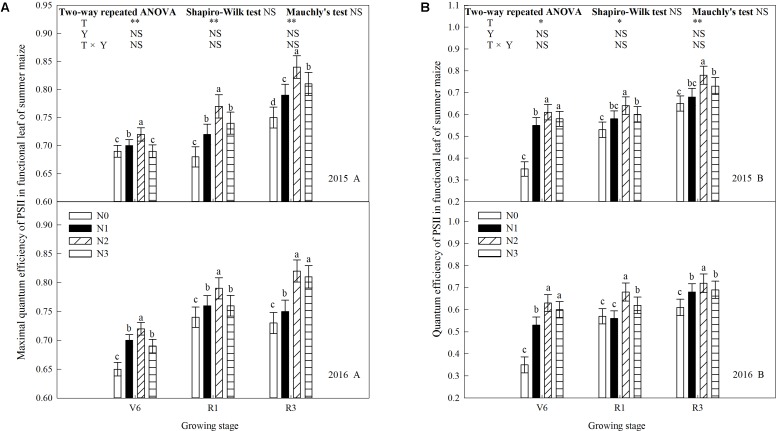
Effect of nitrogen rate on chlorophyll fluorescence in functional leaves of summer maize during growth stages in 2015 and 2016. **(A)** Maximal quantum efficiency, **(B)** quantum efficiency. V6: six-leaf stage, R1: silking stage, R3: milk stage. Nitrogen applied to maize are 0 kg N ha^-1^ (N0), 129 N ha^-1^ (N1), 185 kg N ha^-1^ (N2), and 300 kg N ha^-1^ (N3). Functional leaf: last ligulated leaf at V6, and ear leaf at R1 and R3. Treatments in the same growing stage with different small letters are significantly different at 5% probability level. Error bars are given as SD. NS, not significant, *P* > 0.05. ^∗^, ^∗∗^Significant at the 0.05 and 0.01 probability level, respectively.

N rate affected the maximal quantum efficiency of PSII (*F*_v_/*F*_m_) and the quantum efficiency of PSII (Φ_PSII_) significantly (**Figure 7**). *F*_v_/*F*_m_ and Φ_PSII_ increased during growth stages and increased significantly when N rate increased up to N2 (*P* ≤ 0.05) and the trends of 2 years’ results were similar (*P* > 0.05). *F*_v_/*F*_m_ between N2 and N3 did not differ at R3 in 2016 (*P* > 0.05), and Φ_PSII_ between N2 and N3 did not at V6 (*P* > 0.05). In addition to above, *F*_v_/*F*_m_ and Φ_PSII_ of N3 were significantly lower than that of N2 (*P* ≤ 0.05). Take 2015 as an example, *F*_v_/*F*_m_ of N2 increased by 4.3% (*P* ≤ 0.05), 2.9% (*P* ≤ 0.05), and 4.3% (*P* ≤ 0.05) at V6; 13.2% (*P* ≤ 0.05), 6.9% (*P* ≤ 0.05), and 4.1% (*P* ≤ 0.05) at R1; 12.0% (*P* ≤ 0.05), 6.3% (*P* ≤ 0.05), and 3.7% (*P* ≤ 0.05) at R3 compared with N0, N1, and N3, respectively. Φ_PSII_ of N2 increased by 7.4% (*P* ≤ 0.05), 10.9% (*P* ≤ 0.05), and 5.2% (*P* > 0.05) at V6; 20.8% (*P* ≤ 0.05), 10.3% (*P* ≤ 0.05), and 6.7% (*P* ≤ 0.05) at R1; 20.0% (*P* ≤ 0.05), 14.7% (*P* ≤ 0.05), and 6.8% (*P* ≤ 0.05) at R3 compared with N0, N1, and N3, respectively.

### Chloroplast Form and Configuration

**FIGURE 8:**
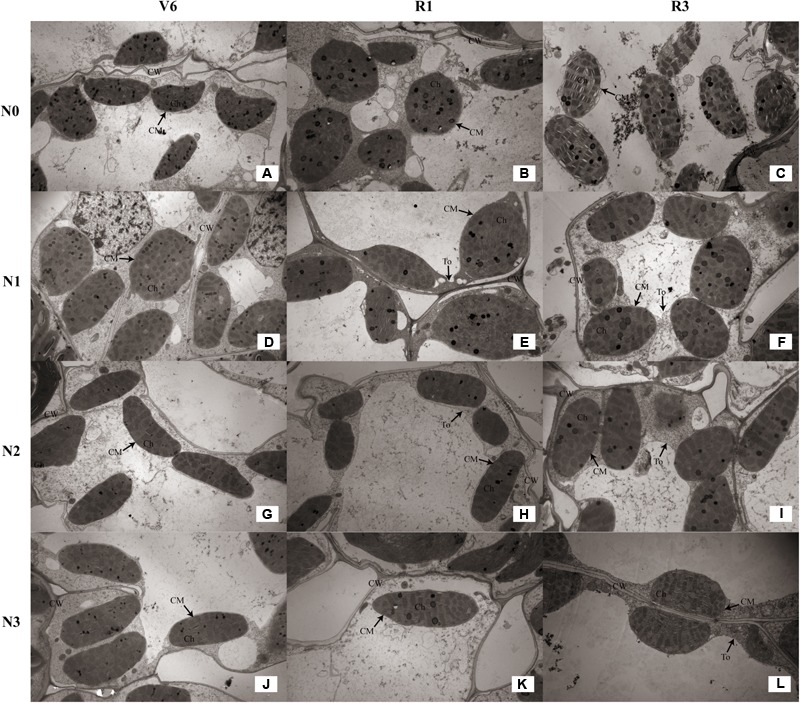
Effect of nitrogen rate on the chloroplast configuration (×10K) during growth stages. **(A–C)** Chloroplast configuration of N0 at V6, R1, and R3, respectively. **(D–F)** Chloroplast configuration of N1 at V6, R1, and R3, respectively. **(G–I)** Chloroplast configuration of N2 at V6, R1, and R3, respectively. **(J–L)** Chloroplast configuration of N3 at V6, R1, and R3, respectively. V6: six-leaf stage, R1: silking stage, R3: milk stage. Nitrogen applied to maize are 0 kg N ha^-1^ (N0), 129 N ha^-1^ (N1), 185 kg N ha^-1^ (N2), and 300 kg N ha^-1^ (N3). Ch: chloroplast, CM: chloroplast membrane, CW: cell wall, To: tonoplast.

As show in **Table 4**, the number of chloroplasts per mesophyll cell of N1 and N1 decreased by 33% (*P* ≤ 0.05) and 9% (*P* ≤ 0.05) at V6; 38% (*P* ≤ 0.05) and 20% (*P* ≤ 0.05) at R1; 27% (*P* ≤ 0.05) and 10% (*P* ≤ 0.05) at R3 compared with N2, respectively. N2 and N3 did not differ (*P* > 0.05). The external of chloroplasts changed from long and oval to elliptical, almost circular or irregular under N0 and N1 (**Figures 8B,E**). Chloroplast morphology was the most damaged under N0 occurred at R3 (**Figure 8C**), and the damage degree increased with growing stage. The length of chloroplasts for N0 and N1 decreased by 14–33% (*P* ≤ 0.05) and 8–22% (*P* ≤ 0.05), and width increased by 17–29% (*P* ≤ 0.05) and 15–18% (*P* ≤ 0.05), respectively, compared with N2. N2 and N3 did not differ in chloroplast size (*P* > 0.05), except length for N3 was higher (*P* ≤ 0.05) at V6.

**Table 4:** Chloroplast ultrastructure characteristics in mesophyll cells of summer maize last ligulated leaf at six-leaf stage (V6), and ear leaves at silking (R1) and milk stage (R3) as affected by N rate.

Growth stage	Treatment	Chloroplast per mesophyll cell	Chloroplast size		Grana per chloroplast	Lamellae per grana
			Length (μm)	Width (μm)		
V6	N0	6.8^c^	4.9^d^	3.3^a^	20.1^c^	16.3^c^
	N1	8.3^b^	5.2^c^	3.1^a^	23.4^b^	21.1^b^
	N2	10.2^a^	5.7^b^	2.7^b^	25.3^a^	25.4^a^
	N3	9.9^a^	6.1^a^	2.7^b^	25.7^a^	25.2^a^
R1	N0	7.8^c^	5.1^c^	4.9^a^	27.6^a^	10.4^c^
	N1	10.1^b^	5.9^b^	4.5^b^	20.8^c^	24.7^b^
	N2	12.6^a^	7.6^a^	3.8^c^	22.4^b^	30.0^a^
	N3	12.5^a^	7.8^a^	3.9^c^	22.1^b^	30.6^a^
R3	N0	9.1^c^	5.2^b^	4.8^a^	28.3^a^	7.2^d^
	N1	11.2^b^	5.7^b^	4.7^a^	21.0^c^	25.5^c^
	N2	12.5^a^	7.9^a^	4.1^b^	22.1^b^	44.3^a^
	N3	12.7^a^	7.8^a^	4.0^b^	21.9^b^	38.1^b^
Shapiro–Wilk test		NS	NS	NS	NS	NS
Levene test		NS	NS	NS	NS	NS
One-way ANOVA						
Treatment		^∗∗^	^∗∗^	^∗∗^	^∗∗^	^∗∗^


*Values followed by a different small letter in the same growth stage are significantly different at P ≤ 0.05 with Duncan’s multiple range test. NS, not significant, P > 0.05. ^∗∗^Significant at the 0.01 probability level. Nitrogen applied to maize are 0 kg N ha^-*1*^ (N0), 129 N ha^-*1*^ (N1), 185 kg N ha^-*1*^ (N2), and 300 kg N ha^-*1*^ (N3).*

### Chloroplast Ultrastructure

**FIGURE 9:**
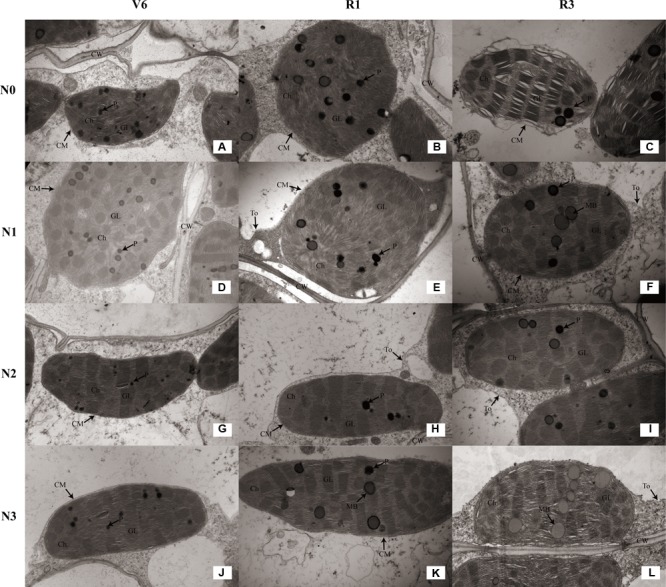
Effect of nitrogen rate on the chloroplast ultrastructure (×25K) during growth stages. **(A–C)** Chloroplast ultrastructure of N0 at V6, R1, and R3, respectively. **(D–F)** Chloroplast ultrastructure of N1 at V6, R1, and R3, respectively. **(G–I)** Chloroplast ultrastructure of N2 at V6, R1, and R3, respectively. **(J–L)** Chloroplast ultrastructure of N3 at V6, R1, and R3, respectively. V6: six-leaf stage, R1: silking stage, R3: milk stage. Nitrogen applied to maize are 0 kg N ha^-1^ (N0), 129 N ha^-1^ (N1), 185 kg N ha^-1^ (N2), and 300 kg N ha^-1^ (N3). Ch: chloroplast, CM: chloroplast membrane, GL: grana lamella, P: particles, CW: cell wall, To: tonoplast, MB: multivesicular body.

As for the N2 and N3 plants, chloroplasts had a complete external envelope and clear boundary, and the thylakoid systems were well-developed (**Figures 8G–L**). The lamella structure pile folds were in order, and both gram lamella and stroma lamellae were arranged compactly and clearly (**Figures 9G–I**). Chloroplasts of N0 and N1 were partially damaged, the external capsule gram lamellae were fuzzy and disordered, the interlayer gap became larger, and multivesicular bodies were occasionally found and most chloroplasts were similarly round and showed external envelope degradation at R3 (**Figures 8A–F, 9A–F**). The chloroplast internal structure was deteriorated, and the numbers of gram lamellae were reduced significantly (*P* ≤ 0.05) to varying degrees. In N0 and N1 treatments, the gram and substrate lamellae were not clearly differentiated, the number of multivesicular body was increased, and individual chloroplasts disintegrated. In N3 treatment, gram and gram lamellae were still well developed and exhibited only partial adventitia fractures. However, the lamellar structure was arranged loosely, and cracks among lamellae were evident and the gram lamellae gradually became twisted (**Figures 9J–L**). The negative effects varied with growth stage. As shown in **Table 4**, the number of lamellae per grana for N2 increased by 56–515% (*P* ≤ 0.05) and 20–74% (*P* ≤ 0.05) compared with N0 and N1, respectively. At V6, the number of grana per chloroplast for N2 was higher (*P* ≤ 0.05) than others, however, that for N0 was higher (*P* ≤ 0.05) at R1 and R3. N2 and N3 did not differ (*P* > 0.05), except lamellae number of N3 was less at R3 (*P* ≤ 0.05).

### Correlation Analysis Between Photosynthetic Characters and Grain Yield

According to correlation analysis, there were positive correlation between grain yield and the parameters of photosynthesis and chlorophyll although someone was not significant (**Table 5**). At V6, chlorophyll SPAD value was significantly correlated with grain yield (*P* ≤ 0.05). At R1, *P*_n_ (*P* ≤ 0.05) and Φ_PSII_ (*P* ≤ 0.01) correlated with grain yield. At R3, LAI, chlorophyll SPAD value and *P*_n_ were positively correlated with grain yield (*P* ≤ 0.05) and the correlation between Φ_PSII_ and yield was extremely significant (*P* ≤ 0.01). As shown in **Table 6**, grain yield correlated positively with the number of chloroplast per cell, grana per chloroplast and lamellae per chloroplast although someone was not significant. There were significantly positive correlations between yield and chloroplast number at R1 (*P* ≤ 0.05) and R3 (*P* ≤ 0.01). The number of lamellae per grana was significantly correlated with yield at R1 and R3 (*P* ≤ 0.05). The number of lamellae per chloroplast correlated significantly with yield during growth stages (*P* ≤ 0.05). Note that the significant positive correlation between yield and grana number appeared at V6 (*P* ≤ 0.05) and negative correlation appeared at R1 (*P* > 0.05) and R3 (*P* ≤ 0.05).

**Table 5:** Correlation coefficients between grain yield of summer maize and the parameters of photosynthesis and chlorophyll fluorescence during growth stages (V6: six-leaf stage; R1: silking stage; R3: milk stage).

Correlation coefficients	V6	R1	R3
LAI	0.853^NS^	0.917^NS^	0.969^∗^
SPAD	0.973^∗^	0.939^NS^	0.976^∗^
*P*_n_	0.898^NS^	0.978^∗^	0.981^∗^
*F*_v_/*F*_m_	0.806^NS^	0.902^NS^	0.851^NS^
Φ_PSII_	0.825^NS^	0.991^∗∗^	0.993^∗∗^


*NS, not significant, P > 0.05. ^∗^, ^∗∗^Significant at the 0.05 and 0.01 probability level, respectively.*

**Table 6:** Correlation coefficients between grain yield of summer maize and chloroplast ultrastructures during growth stages (V6: six-leaf stage; R1: silking stage; R3: milk stage).

Correlation coefficients	V6	R1	R3
Chloroplast per mesophyll cell	0.931^NS^	0.956^∗^	0.995^∗∗^
Grana per chloroplast	0.956^∗^	-0.917^NS^	-0.962^∗^
Lamellae per grana	0.920^NS^	0.985^∗^	0.968^∗^
Lamellae per chloroplast	0.974^∗^	0.955^∗^	0.962^∗^


*NS, not significant, P > 0.05. ^∗^, ^∗∗^Significant at the 0.05 and 0.01 probability level, respectively.*

## Discussion

N is the necessary element for crop growth and development, and also is the limitation to stable and high grain yield (Uhart and Andrade, 1995; Kamara et al., 2003). In the NCP, local farmers usually applied excessive N fertilizer, which led to waste of resources and pollution instead of higher yield (Liu et al., 2003). Previous research proved that 185 kg N ha^-1^ was the appropriate level in this region (Jin et al., 2012). In this study, the average grain yield of N2 increased significantly by 30 and 9% compared with N0 and N1, however, N2 and N3 did not differ, which agreed with the previous reports (Lemcoff and Loomis, 1994; Liu et al., 2017). According to yield components, ears per hectare, kernels per ear and TKW had different levels of increase with increase N rate. There was no significant difference in kernels per ear among N1, N2, and N3, which indicate that kernels per ear is insensitive to N rate. N0 treatment not only impair grain-filling and kernel development but also lead ear abortion, which are consistent with the previous reports (Monneveux et al., 2005). Note that a lower TKW under N3 appeared in 2016. The reason may be that the excessive N delay crop maturation and hinder nutrition transport from vegetative organ to reproductive organ at later growing stage (Schröder et al., 2000; Jin et al., 2012). The key to high grain yield of summer maize is to force ears per hectare, kernels per ear and TKW coordinate more.

N had important effects on photosynthetic characteristics (Sinclair and Horie, 1989; Makino and Osmond, 1991; Sade et al., 2017). LAI reflected the nutritional status and potential photosynthetic area of summer maize (Duncan, 1971; Gitelson et al., 2014). In this study, LAI increased with N rate, however, N2 and N3 did not differ, which coincides with the previous study (Liu et al., 2017). The grain yield was significantly and positively correlated with the LAI at R3, that is, it is important for grain yield to maintain a high level of LAI at later growing stage. The chlorophyll SPAD value was used to measure relative chlorophyll content in leaf (Costa et al., 2001; Martínez and Guiamet, 2004). In this study, the more N fertilizer will help maintain high chlorophyll SPAD value at later growing stage, however, N2 and N3 did not differ at early growing stage. According to correlation analysis, grain yield was significantly and positively correlated with the chlorophyll SPAD value at V6 and R3. At early growing stage, the higher chlorophyll SPAD value promote the using efficiency of light and the growth and development of vegetative organs. At later growing stage, maintaining higher chlorophyll SPAD value promote photosynthesis which help grain-filling. The great N state improve gas exchange parameters significantly. In this study, *P*_n_ of N2 increased by 101–113% and 36–37% compared with N0 and N1, respectively. *T*_r_ and *G*_s_ increased, and *C*_i_ decreased with N rate increased up to N2. According to previous studies (Sadras and Milroy, 1996; Hura et al., 2007; Anjum et al., 2011), N0 and N1 treatments limit photosynthesis resulting from both stomatal limitation (gas exchange between functional leaf and environment is limited) and non-stomatal limitation (carboxylation is impaired, that is, the photosynthetic apparatus are destroyed). In comparison with N2, *T*_r_ and *G*_s_ increased by 31–66% and 7–17%, respectively, under N3, that is, the loss of water is higher. The grain yield of summer maize had significantly positive correlation with *P*_n_ at R1 and R3. This suggest that one of the keys to high grain yield is increasing photosynthetic rate and extending peak-hours of photosynthesis (Tollenaar and Lee, 2002). *F*_v_/*F*_m_ and Φ_PSII_ are indicators to evaluate the performance of photosynthesis (Genty et al., 1989; Wagle et al., 2016; Su et al., 2017). In this study, *F*_v_/*F*_m_ of N2 increased by 4–13%, 3–7%, and 4%, and Φ_PSII_ of N2 increased by 7–21%, 10–15%, and 5–7% compared to N0, N1 and N3, respectively. This indicate that appropriate N rate improve the performance of PSII. The correlation between grain yield and *F*_v_/*F*_m_ did not reach significant level during the growing stages. The grain yield had extremely significant and positive correlation with Φ_PSII_ at R1 and R3, which suggest that the performance of PSII have significant effects on grain yield, especially at reproductive growth stage (Lu and Zhang, 2000). The above photosynthetic characteristics affected dry matter weight. During the period from V6 to R6, dry matter weight of N0 was lower than the others and the gap between N0 and N2 enlarged with growing stage, which is consistent with previous research (Binder et al., 2000; Ciampitti and Vyn, 2012).

The photosynthetic characteristics are close to chloroplast configuration and ultrastructure (Giles et al., 1976; Niki et al., 1978). Chloroplast is the main site that produce ROS under stress (Xu et al., 2006). Previous studies indicated that chill stress led to the rupture of chloroplast membrane, and the drought and shade would destroy thylakoid membrane (Giles et al., 1976; Niki et al., 1978; Weston et al., 2000). Normal chloroplast configuration and structure are important to photosynthesis. In this study, N0 and N1 treatments caused the chloroplast arranged scattered and ultrastructural damage, and membrane of chloroplast and thylakoid had different degrees of injury, meanwhile, lamellae per grana significantly decreased. The structural damage inevitably result in functional disorder manifesting as decreases in chlorophyll SPAD value (Zhang et al., 2009) and parameters of gas exchange and fluorescence (Giles et al., 1974, 1976). And the damage degree of chloroplast morphology and ultrastructure increased with growing stage, which may result in faster aging at later growing stage compared with those maize under N2 and N3 treatments (Kołodziejek et al., 2003). The result was a reduction in grain yield. N3 treatment led to increase in the number of osmium granule and vesicle, and the gap between lamellae enlarged, which may be the reason for decreases in photosynthetic rate and quantum efficiency (Stirling et al., 1991; Sowiński et al., 2005). The well-developed chloroplast characterized by large grana, more lamellae and compact structure is important for photosynthesis and grain yield.

## Conclusion

In this study, 185 kg N ha^-1^ is the appropriate application rate for grain yield, photosynthesis and chloroplast ultrastructure. The changes in N rate result in the difference in photosynthetic characteristics and chloroplast ultrastructure of field-grown maize, which is one of the reasons for yield gap between different N treatments. The leaf area index and chlorophyll SPAD value increase with nitrogen rate, and the gap between treatments is greater in later growing stage. The chloroplasts have a complete external envelope and clear boundary, and the thylakoid systems are well-developed, and lamellae of both gram and stroma are arranged compactly and clearly under N2. N0 (0 kg N ha^-1^) and N1 (129 kg N ha^-1^) treatments result in both stomatal limitation and non-stomatal restriction, and the damage to chloroplast – from the inside out. N3 (300 kg N ha^-1^) treatment increases the loss of water instead of raising photosynthetic rate, and lead to more loose lamellae structure and greater gap between lamellae.

## Author Contributions

ZL carried out the measurements, data analysis, and drafted the manuscript. JZ designed the experiments. JG, FG, PL, BZ, and JZ made substantial contributions to conception, and critically revised the manuscript.

## Conflict of Interest Statement

The authors declare that the research was conducted in the absence of any commercial or financial relationships that could be construed as a potential conflict of interest.
